# Case Report: Compound heterozygous mutation comprising p.Pro31Leu and exons 1–3 ins/del variants in *CYP21A2* causes non-classical congenital adrenal hyperplasia in a Chinese girl

**DOI:** 10.3389/fped.2026.1778805

**Published:** 2026-03-31

**Authors:** Nan Li, Chao Lu, Harvest F. Gu, Xiaofei An

**Affiliations:** 1 Department of Endocrinology, Jiangsu Province Hospital of Chinese Medicine, The Affiliated Hospital of Nanjing University of Chinese Medicine, Nanjing, Jiangsu, China; 2 Department of Radiology, Jiangsu Province Hospital of Chinese Medicine, The Affiliated Hospital of Nanjing University of Chinese Medicine, Nanjing, Jiangsu, China; 3 Laboratory of Molecular Medicine, School of Basic Medicine and Clinical Pharmacy, China Pharmaceutical University, Nanjing, Jiangsu, China; 4 College of Pharmacy, Qilu Medical University, Zibo, Shandong, China

**Keywords:** compound heterozygous mutation, *CPY21A2*, genetic analysis, non-classic form of congenital adrenal hyperplasia, steroid 21-hydroxylase deficiency

## Abstract

Congenital adrenal hyperplasia (CAH) is caused by variants in the *CYP21A2* gene and subsequently results in 21-hydroxylase deficiency. The non-classic form of CAH (NCCAH) often occurs in late puberty or in young adults due to a mild excess in postnatal androgen synthesis. This case report presents a case of a Chinese girl diagnosed with NCCAH. Clinical examinations and adrenal computed tomography were conducted in accordance with the standards and guidelines. Further genetic analysis was performed using PCR, next-generation sequencing, and multiplex ligation-dependent probe amplification. Clinical examinations demonstrated that the patient had amenorrhea, hyperandrogenism, insulin resistance, adrenal hyperplasia, and a masculine vulva, while polycystic ovary syndrome was excluded. Genetic analysis identified a compound heterozygous mutation comprising the c.92C>T p.Pro31Leu and exons 1–3 del variants in the *CYP21A2* gene, while the exons 1–3 ins/del variant mainly caused the lack of CYP21A2 function. Herein, we report a Chinese girl with NCCAH due to a heterozygous mutation comprising the p.Pro31Leu and exons 1–3 ins/del variants in *CYP21A2*.

## 1 Introduction

Congenital adrenal hyperplasia (CAH) is a group of autosomal recessive disorders characterized by impaired cortisol synthesis. Accumulating evidence has demonstrated that the majority of CAH cases (nearly 95%) are caused by mutations in the cytochrome P450 family 21 subfamily A member 2 (*CYP21A2*) gene and subsequently result in 21-hydroxylase deficiency (21-OHD, OMIM: 201910) (1–5). Clinical manifestations of 21-OHD are classified into classic salt-wasting (SW), classic simple virilizing (SV), and non-classic (NC) forms. Non-classical congenital adrenal hyperplasia (NCCAH) is a milder, later-onset form of CAH, a group of inherited disorders affecting the adrenal glands' ability to produce essential hormones. NCCAH often occurs in late puberty or in young adults due to the mild excess in postnatal androgen synthesis (6). In general, subjects with NCCAH are affected by milder allelic variants, while the associated alleles may encode the enzymes with residual activity of 20%–50%. Therefore, adolescent girls with NCCAH typically have normal basal cortisol and aldosterone production but mildly elevated levels of adrenal androgens (6, 7). Adult women, however, often present with hirsutism, oligomenorrhea, acne, and subnormal fertility, which may be misdiagnosed as polycystic ovarian syndrome (PCOS) (8). Furthermore, NCCAH is a late-onset and milder form of 21-hydroxylase deficiency marked by adrenal androgen excess. The psychoneuro-social effects of NCCAH are heterogeneous and shaped by sex, age, and clinical presentation (9).

The record of more than 230 genetic variants in the *CYP21A2* gene has been updated (3, 10, 11). These variants are associated either with severe salt-wasting or simple virilizing phenotypes or with milder nonclassical phenotypes. Not all of these genetic variants in *CYP21A2*, however, are pathogenic. Some have been characterized biochemically, while the clinical consequences of others still remain unknown (11). Hou et al. previously demonstrated a correlation between the mutation frequency of the *CYP21A2* gene and the 21-OHD phenotype–genotype in patients from southern China (12). Lan et al. recently suggested that targeted capture combined with long-read sequencing may be an integrated approach for detecting *CYP21A2* mutations based upon the analyses of 67 patients with 21-OHD (13). Zhao et al. demonstrated that there is a high incidence (57.4%) of the SV form among 21-OHD patients who harbor the p.Pro31Leu variant in Chinese populations (14). Herein, we report the clinical diagnosis and genetic analysis of a Chinese girl with NCCAH due to the p.Pro31Leu and exons 1–3 ins/del variants in the *CYP21A2* gene. The diagnosis, including a clinical observation, laboratory tests, adrenal computed tomography, and genetic analysis, is discussed.

## 2 Case presentation

### 2.1 Patient

An 18-year-old girl was initially referred to the Department of Endocrinology in our hospital due to amenorrhea. Prior to this, she had a normal menstrual cycle of 28–30 days, with menarche at the age of 12. She denied any sexual history. A couple of years ago, she had an incision and drainage of a perianal abscess because she had been suffering from Crohn's disease. Since then, she had been treated with mesalazine tablets to relieve intestinal inflammation. Currently, she has a body mass index of 28.5 kg/m^2^ (height was 156 cm and body weight was 69.3 kg). In the last 2 years, her body weight has increased by nearly 20 kg.

### 2.2 Clinical examination

The patient was then admitted following a clinical observation, laboratory tests, adrenal computed tomography, and genetic analysis in our Department of Endocrinology. A clinical observation and physical examination were initially conducted, with laboratory tests and adrenal computed tomography following. All of these were conducted in accordance with the standards and guidelines of our hospital.

### 2.3 Genetic analysis

Further genetic analysis was performed using PCR, next-generation sequencing (NGS), and multiplex ligation-dependent probe amplification (MLPA). First, peripheral blood samples (4 mL each) were taken from the proband and her parent. The genomic DNA was then extracted from peripheral blood leukocytes. The DNA library was then hybridized with target enrichment capture probes according to the MyGenostics GenCap standard enrichment protocol (http://www.mygenostics.com). Finally, the hybrid products were purified and sequenced using an Illumina HiSeq2000 PEI101 (Illumina, San Diego, USA). Based upon the information from the OMIM (Online Mendelian Inheritance in Man) database, a total of 280 genes, including *CYP21A2*, were selected for gene sequencing analysis (a list of the genes included in the genetic sequencing analysis is presented in the Supplementary Material).

### 2.4 Bioinformatics

We evaluated the biological effects of *CPY21A2* variants using the SWISS-MODEL protein molecular modeling program (https://swissmodel.expasy.org/) and AlphaFold (https://alphafold.ebi.ac.uk/) to perform protein structure analyses based on the UniProtKB database (15–17).

## 3 Results

### 3.1 Clinical examination

**Figure 1:**
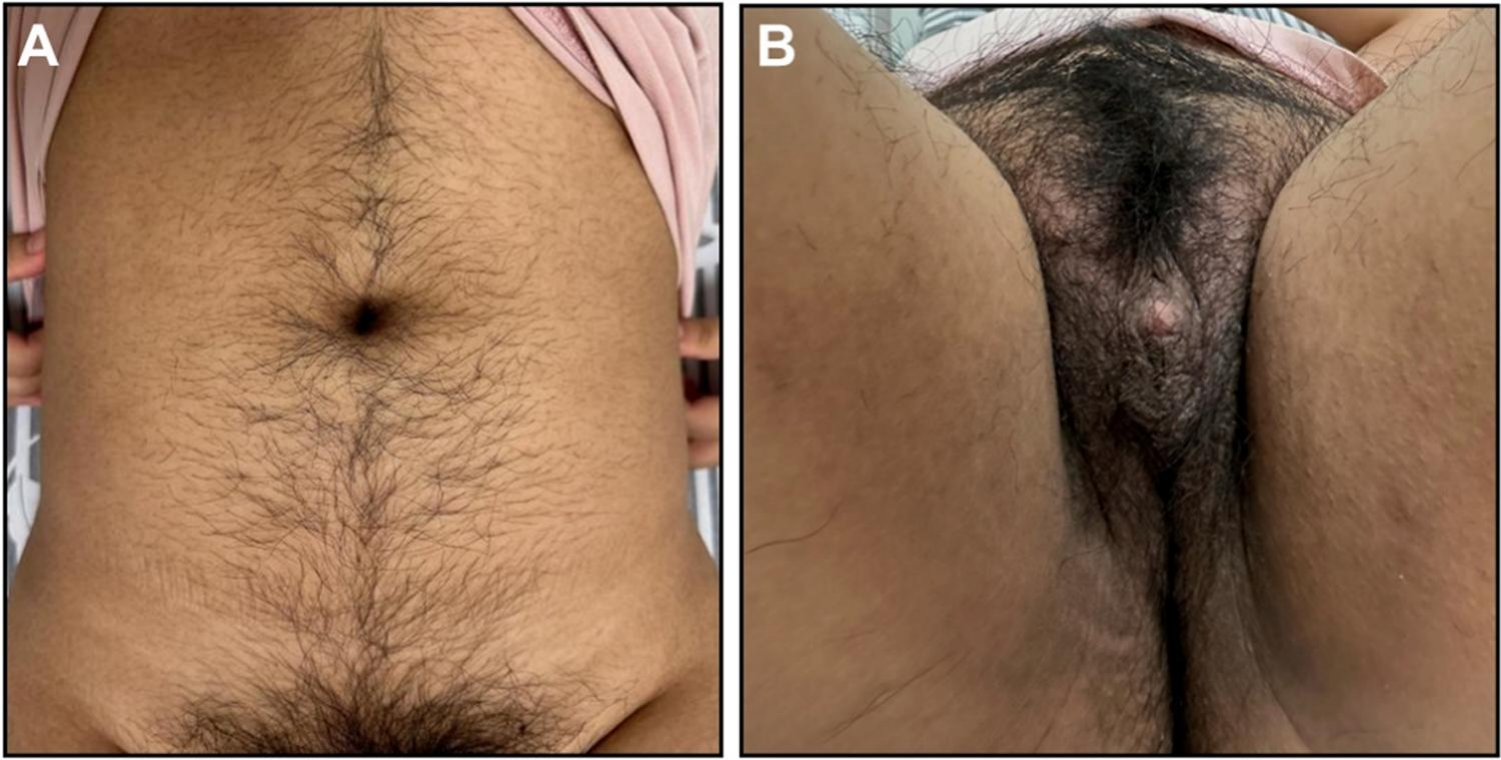
The abdominal hirsutism and vulva clitoromegaly of the proband.

The clinical observation found that her skin looked darker and her face had scattered acne. Furthermore, there was acanthosis nigricans on her nape. The axillae displayed distinctive purple-colored patterns, which were non-blanchable and appeared to be of vascular or pigmented origin. These patterns were bilateral and symmetrical, with a reticular or mottled appearance. As seen in Figure 1A, there was excessive hair on her entire abdomen, perineum, and lower limbs (Ferriman–Gallwey hair score = 12). Figure 1B illustrates pigmentation and heavy hair on her perineum as well as vulva clitoromegaly. A further clinical examination as an outpatient found that she had a testosterone (T) level of 135.94 ng/dL (normal, 0.00–75.00 ng/mL), fasting insulin level of 48.67 µIU/mL (1.90–23.00 µIU/mL), and fasting C-peptide level of 5.28 ng/mL (normal, 0.78–5.19 ng/mL).

### 3.2 Laboratory tests

The data from the laboratory test are summarized in Table 1. The patient’s electrolyte levels were as follows: potassium 3.68 mmol/L (normal, 3.50–5.10 mmol/L) and sodium 138.3 mmol/L (normal, 136.0–146.0 mmol/L). However, her 17-hydroxyprogesterone level was found to be much higher than normal at 30.00 ng/mL (normal, 0.00–1.05 ng/mL). Her dehydroepiandrosterone level was 438.1 μg/dL (normal, 56.2–511.7 μg/dL) and her T level was 189.16 ng/dL (normal, 0.00–75.00 ng/mL). Moreover, the patient’s serum cortisol rhythms (at 00:00 h, 08:00 h, and 16:00 h) were 2.4, 10.8, and 4.9 μg/dL, respectively (normal morning, 6.7–22.6 μg/dL; afternoon <10 μg/dL), while her adrenocorticotropic hormone (ACTH) rhythms were 3.44, 68.18, and 19.14 pg/m (normal morning, 7.20–63.3 pg/mL). Furthermore, her 24 h urinary free cortisol level was 93 μg/24 h (normal, 58–403 μg/24 h). The standardized 75 g oral glucose tolerance test indicated that her blood glucose level was normal, but insulin secretion exceeded the basic value 10-fold. The insulin resistance index calculation was performed and her Homeostatic Model Assessment of Insulin Resistance score was 2.45. The patient’s C-peptide level was 1.64 (ng/mL) at 0 min and 11.87 (ng/mL) at 120 min. In addition, the proband’s blood pressure was normal at 118/80 mmHg.

**Table 1:** Clinical examination data.

Laboratory tests	17-OHP (ng/mL)	24.70
	T (ng/dL)	189.16
	DHEAS (µg/dL)	438.1
	FSH (mIU/mL)	4.06
	E2 (mIU/mL)	<20
	P (ng/mL)	5.73
	PRL (ng/mL)	11.52
	Na^+^ (mmol/L)	138.3
	K^+^ (mmol/L)	3.68
	Cortisol (μg/dL) 8:00/16:00/24:00	10.8/4.9/2.4
	ACTH (pg/mL) 8:00/16:00/24:00	68.18/19.14/3.44
	24 h urinary free cortisol (μg/24 h)	93.0
OGTT	Blood glucose (mmol/L) 0 min/120 min	3.96/6.63
	Insulin (µIU/mL) 0 min/120 min	13.94/193.53
	C-peptide (ng/mL) 0 min/120 min	1.64/11.87

ACTH, adrenocorticotropic hormone; DHEAS, dehydroepiandrosterone; E2, estradiol; FSH, follicle-stimulating hormone; P, progesterone; PRL, prolactin; T, testosterone; 17-OHP, 17-hydroxyprogesterone; OGTT, the standardized 75-g oral glucose tolerance test.

### 3.3 Tomographic analysis of the adrenal glands

**Figure 2:**
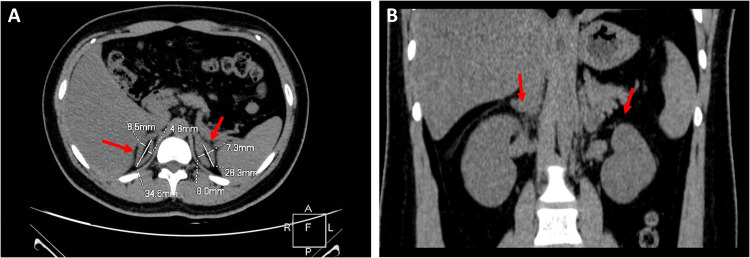
Adrenal computed tomography images. As demonstrated by the transverse **(A)** and coronal **(B)** images, the bilateral adrenal volumes in the proband were increased.

To evaluate the size of her adrenal glands, adrenal computed tomography was performed alongside a gynecological (transrectal) color Doppler ultrasound. In Figure 2, the transverse (**A**) and coronal (**B**) images show that the bilateral adrenal glands of the patient were large and the size of the left and right adrenal glands are presented in mm. No nodules were observed in the glands. The volume of the patient’s left and right adrenal glands was 8.9 cc and 9.6 cc, respectively (Supplementary Figures S1a,b). In addition, her uterus was in the anterior position, 31.9 mm × 26.4 mm × 21.1 mm in size with an endometrial thickness of 2.3 mm, and her cervix was normally shaped. The left and right ovaries were 31.1 mm × 20.05 mm and 25.0 mm × 20.4 mm in size, respectively. No abnormalities in the uterus and adnexa were seen.

### 3.4 Identification of genetic variants in *CPY21A2*

**Figure 3:**
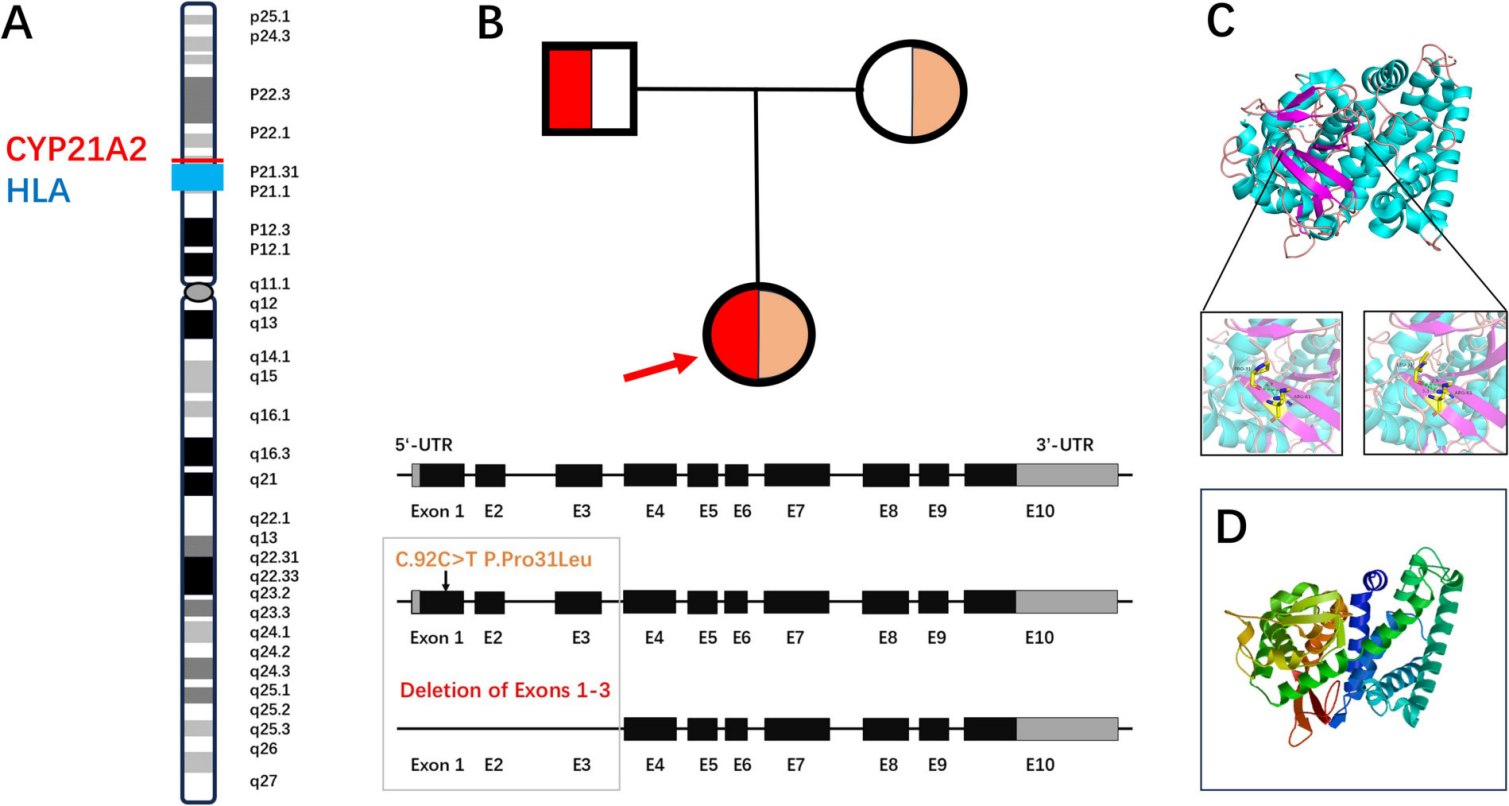
A diagrammatic sketch of NCCAH due to a compound heterozygous mutation comprising the exons 1–3 ins/del and p.Pro31Leu variants in the *CPY21A2* gene. **(A)** The *CYP21A2* gene is localized in chromosome 6p21.33, which is close to the HLA region. The gene encodes the CYP21A2 protein and consists of 10 exons. In the first and 10th exons, there is one untranslated region. **(B)** The family pedigree demonstrates that the daughter (proband) has NCCAH due to a heterozygous mutation, i.e., deletion of exons 1–3 and c.92C>T p.Pro31Leu in the first exon of the *CPY21A2* gene, while her mother is a heterozygous carrier of p.Pro31Leu. Her father is the carrier of the deletion of exons 1–3. The locations of the p.Pro31Leu and exons 1–3 ins/del variants are indicated in the gene structure. **(C,D)** The structural changes in the protein resulting from the p.Pro31Leu mutation and exons 1–3 ins/del are respectively illustrated.

The genetic analysis identified a compound heterozygous mutation comprising the p.Pro31Leu and exons 1–3 ins/del variants in the *CPY21A2* gene in this patient with NCCAH. As shown in Figure 3A, the *CYP21A2* gene is localized in chromosome 6p21.33, which is close to the human leukocyte antigen (HLA) region. The gene encodes the CYP21A2 protein and consists of 10 exons. In the first and 10th exons, there is one untranslated region. Based on the genetic analysis using NGS and MLPA, we found that the proband had a heterozygous mutation comprising exons 1–3 del and c.92C>T p.Pro31Leu in the first exon of the *CPY21A2* gene, while her mother was a heterozygous carrier of c.92C>T p.Pro31Leu. In her father, the deletion of exons 1–3 in *CYP21A2* was detectable. Locations of p.Pro31Leu and exons 1–3 ins/del variants were indicated in the gene structure (Figure 3B). Furthermore, the CPY21A2 protein had 495 amino acids (AA) and a mass of 56,001 Da. Bioinformatic tools were used to predict the biological function of CPY21A2 due to the variants. The structural changes in the protein resulting from the p.Pro31Leu mutation and exons 1–3 ins/del are illustrated in Figures 3C,D, respectively.

## 4 Discussion

We identified a heterozygous mutation of the *CYP21A2* gene, i.e., c.92C>T p.Pro31Leu and exons 1–3 ins/del, in a Chinese girl with NCCAH. As previously reported, the *CYP21A2* gene is localized at chromosome 6p21.33, near the HLA region. A pseudogene (*CYP21A1P*) for *CYP21A2* is located approximately 30 kb away. The sequence homology between *CYP21A2* and this pseudogene is approximately 98% in exons and 96% in introns. Due to the high degree of similarity between these two genes, a high frequency of recombination events in their genomic region can occur during meiosis, resulting in varying degrees of CYP21A2-related enzymatic deficiency (3, 18, 19). There is evidence that approximately 95% of CYP21A2 disease-causing mutations are variants or deletions derived from *CYP21A1P* through recombination (3, 4, 10, 20). The majority of patients with CAH, including those with NCCAH, are carriers of compound heterozygotes with different mutations on each allele and a phenotype associated with a milder gene defect (6). In our study, we identified a compound heterozygous mutation comprising the p.Pro31Leu and exons 1–3 ins/del variants in the proband. These variants were each derived from her parents.

p.Pro21Leu is a missense mutation in exon 1 of the *CYP21A2* gene, resulting in the substitution of proline with leucine at position 31. This mutation is located in the signal peptide region and may impair the proper processing and trafficking of the enzyme precursor, thereby reducing the activity of the mature enzyme, although not completely abolishing it. As a result, patients typically retain partial 21-hydroxylase activity, which can lead to hormonal imbalances during critical periods of growth and development. Clinically, this mutation is primarily associated with 21-OHD (6–8). The full coding region of exons 1–10 in the *CYP21A2* gene spans approximately 3.1 kb. The deletion of exons 1–3 totals 0.564 kb (564 bp, with exon 1 = 219 bp, exon 2 = 159 bp, and exon 3 = 186 bp). This deletion constitutes a large genomic deletion and usually results in complete loss of 21-hydroxylase activity (<1%), leading to the classical salt-wasting form of CAH. However, when this deletion occurs in compound heterozygosity with p.Pro31Leu, the phenotype may be attenuated and present as the non-classical form. In affected children, the main manifestations include accelerated bone age, rapid growth velocity, and compromised final adult height, along with premature pubarche (9). In adolescent females, as reported in this case, the primary symptoms include oligomenorrhea or amenorrhea, hirsutism, infertility, and acne, which are often misdiagnosed as PCOS. These patients and their parents may experience significant psychological stress, particularly concerning physical appearance and future fertility, which should be carefully addressed in clinical practice.

Based on the diagnosis and treatment of this clinical case, we suggest that in clinical practice, women with irregular menstruation should be routinely evaluated for NCCAH. This is due to genetic variations in the *CYP21A2* gene, such as exons 1–3 ins/del, which result in defective enzyme activity. Consequently, 21-hydroxylase activity is reduced, leading to decreased cortisol synthesis. Through a negative feedback mechanism, this triggers increased ACTH secretion from the pituitary gland, stimulating adrenal cortical cell hyperplasia (20). The abnormal accumulation of adrenal synthetic substrates then typically results in bypass metabolic hyperactivity, namely hyperandrogenism (9). Evidence has demonstrated that women with NCCAH frequently exhibit gonadal dysfunction, presenting as amenorrhea, anovulatory menstruation, oligomenorrhea, or infertility. Only 2.2% to 4.0% of adult women with NCCAH show irregular menstruation, while 56% of girls aged 10–19 experience oligomenorrhea and 9% have primary amenorrhea (6, 21). Therefore, in clinical settings, women with menstrual irregularities should be systematically assessed for NCCAH. Additionally, some patients with NCCAH may develop PCOS, primarily due to adrenal-derived androgens interfering with gonadotropin release and directly acting on the ovaries, ultimately leading to the formation of androgen-secreting cysts. Approximately 10% of PCOS cases can be attributed to NCCAH (22). Hirsutism is also common among women with NCCAH, with 71% scoring over 8 points on the Ferriman–Gallwey scale, and 54% scoring over 15 points (23). Thus, the presence of any clinical manifestations of hyperandrogenism in adolescent women should raise suspicion for NCCAH.

## 5 Conclusions

We have reported a Chinese girl with NCCAH due to a compound heterozygous mutation comprising the p.Pro31Leu and exons 1–3 ins/del variants in the *CYP21A2* gene. We suggest that comprehensive clinical tests and a genetic analysis of *CYP21A2* are essential to avoid a misdiagnosis of NCCAH, while women with irregular menstruation should be routinely evaluated for NCCAH.

## Data Availability

The datasets presented in this article are not readily available because of ethical and privacy restrictions. Requests to access the datasets should be directed to the corresponding author.
