# Supplementary material for: Case Report: Compound heterozygous mutation comprising p.Pro31Leu and exons 1–3 ins/del variants in CYP21A2 causes non-classical congenital adrenal hyperplasia in a Chinese girl
Source: Front Pediatr. 2026 Mar 31;14:1778805. doi: 10.3389/fped.2026.1778805 (PMC13076340; doi:10.3389/fped.2026.1778805)
Supplement: Supplementary file 2 [file Datasheet2.pdf]

Table 1 Data from clinical examination

Laboratory test	17-OH (ng/mL)	24.70
	T (ng/dL)	189.16
	DHEAS (ug/dL)	438.1
	FSH (mIU/mL)	4.06
	E2 (mIU/mL)	<20
	P (ng/mL)	5.73
	PRL (ng/mL)	11.52
	Na^+ (mmol/L)	138.3
	K^+ (mmol/L)	3.68
	Cortisol (μg/dL) 8:00/16:00/24:00	10.8/4.9/2.4
	ACTH (pg/mL) 8:00/16:00/24:00	68.18/19.14/3.44
	24h urinary free cortisol (μg/24 h)	93.0
OGTT	Blood glucose (mmol/L) 0 min/120 min	3.96/6.63
	Insulin (uIU/mL) 0 min/120 min	13.94/193.53
	C-peptide (ng/mL) 0 min/120 min	1.64/11.87

ACTH: adrenocorticotropic hormone; DHEAS: dehydroepiandrosterone; E2: Estradiol; FSH: follicle stimulating hormone; P: progesterone; PRL: prolactin; T: testosterone; 17-OH: 17-hydroxyprogesterone. OGTT: The standardized 75-g oral glucose tolerance test.
