# Supplementary material for: Case Report: Compound heterozygous mutation comprising p.Pro31Leu and exons 1–3 ins/del variants in CYP21A2 causes non-classical congenital adrenal hyperplasia in a Chinese girl
Source: Front Pediatr. 2026 Mar 31;14:1778805. doi: 10.3389/fped.2026.1778805 (PMC13076340; doi:10.3389/fped.2026.1778805)
Supplement: Supplementary file 3 [file Table1.docx]

**Supplemental Figure 1**

A
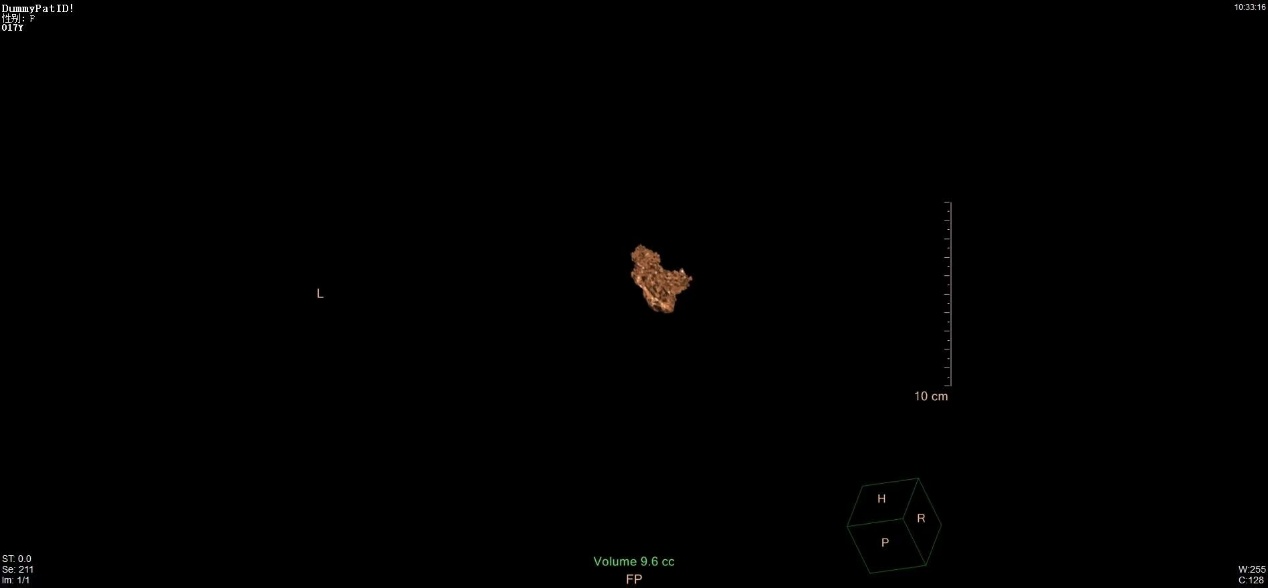


B
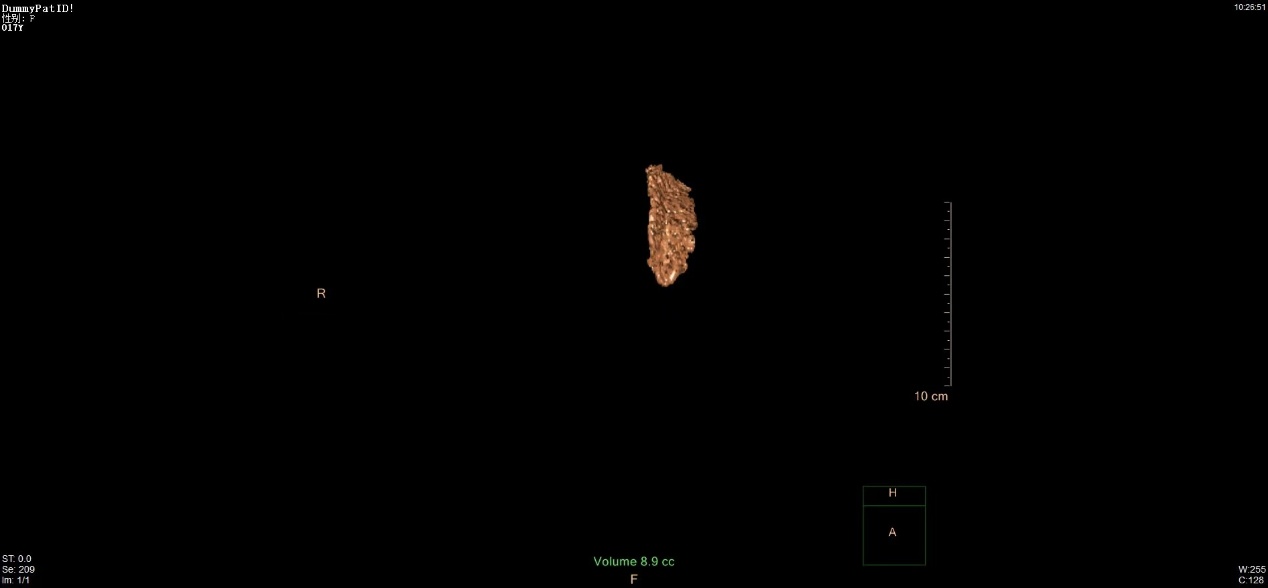


Note: The images above are generated by our hospital imaging equipment, where L actually indicates the patient's right side and R indicates the left side. Additionally, due to the scaling, the left adrenal gland volume is 8.9 cc, although it appears larger; the right adrenal gland volume is 9.6 cc.
