# Supplementary material for: Case Report: Compound heterozygous mutation comprising p.Pro31Leu and exons 1–3 ins/del variants in CYP21A2 causes non-classical congenital adrenal hyperplasia in a Chinese girl
Source: Front Pediatr. 2026 Mar 31;14:1778805. doi: 10.3389/fped.2026.1778805 (PMC13076340; doi:10.3389/fped.2026.1778805)
Supplement: Supplementary file 4 [file Table2.docx]

**Table 1 Data from clinical examination**

| Laboratory test | 17-OH (ng/mL) | 24.70 |
| --- | --- | --- |
|  | T (ng/dL) | 189.16 |
|  | DHEAS (ug/dL) | 438.1 |
|  | FSH (mIU/mL) | 4.06 |
|  | E2 (mIU/mL) | <20 |
|  | P (ng/mL) | 5.73 |
|  | PRL (ng/mL) | 11.52 |
|  | Na^+^ (mmol/L) | 138.3 |
|  | K^+^ (mmol/L) | 3.68 |
|  | Cortisol (μg/dL) 8:00/16:00/24:00 | 10.8/4.9/2.4 |
|  | ACTH (pg/mL) 8:00/16:00/24:00 | 68.18/19.14/3.44 |
|  | 24h urinary free cortisol (μg/24 h) | 93.0 |
| OGTT | Blood glucose (mmol/L) 0 min/120 min | 3.96/6.63 |
|  | Insulin (uIU/mL) 0 min/120 min | 13.94/193.53 |
|  | C-peptide (ng/mL) 0 min/120 min | 1.64/11.87 |

ACTH: adrenocorticotropic hormone; DHEAS: dehydroepiandrosterone; E2: Estradiol; FSH: follicle stimulating hormone; P: progesterone; PRL: prolactin; T: testosterone; 17-OH: 17-hydroxyprogesterone. OGTT: The standardized 75-g oral glucose tolerance test.
